# Supplementary material for: Assessment of Diet Quality and Adherence to Dietary Guidelines in Gastrointestinal Cancer Survivors: A Cross-Sectional Study
Source: Nutrients. 2020 Jul 27;12(8):2232. doi: 10.3390/nu12082232 (PMC7468856; doi:10.3390/nu12082232)
Supplement: Supplementary file 1 [file nutrients-12-02232-s001.pdf]

Table S1. LLD score-2018 and food components in GI cancers survivors and reference population, Lifelines 2006-2009

	GI cancers survivors (n=307)	Reference population (n=3070)	
	Mean (SD)	Mean (SD)	(P-value)2
LLDS	25.71 (5.92)	25.92 (5.91)	0.35
Desired food component			
Vegetables	102.51 (54.93)	107.39 (57.51)	0.05
Fruits	180.85 (115.53)	173.06 (126.94)	0.45
Whole grain products	101.69 (51.57)	109.28 (53.39)	0.11
Legumes and Nuts	24.06 (26.49)	22.85 (22.26)	0.72
Fish	17.67 (17.81)	14.52 (13.56)	0.80
Oil and soft margarine	15.03 (16.40)	16.07 (16.99)	0.71
Unsweetened dairy	210.25 (193.76)	194.41 (157.37)	0.91
Coffee	440.14 (226.37)	460.63 (234.53)	0.46
Tea	266.80 (227.80)	251.41 (230.31)	0.00
Undesired food component			
Red and Processed meat	61.54 (33.46)	62.65 (32.08)	0.09
Butter, hard margarine	31.26 (24.39)	29.81 (23.53)	0.33
Sugar sweetened beverages	109.51 (157.24)	96.13 (133.16)	0.26
Daily alcohol intake	7.49 (8.88)	7.77 (9.09)	0.41

[1]All food components are presented in mean intake in grams per day.

[2]Results from a multivariate linear regression adjusted for age, sex, smoking, BMI, education level and physical activity

Abbreviations: CI, confidence interval; LLD, life Line diet; MD, mean difference; SD, standard deviation.

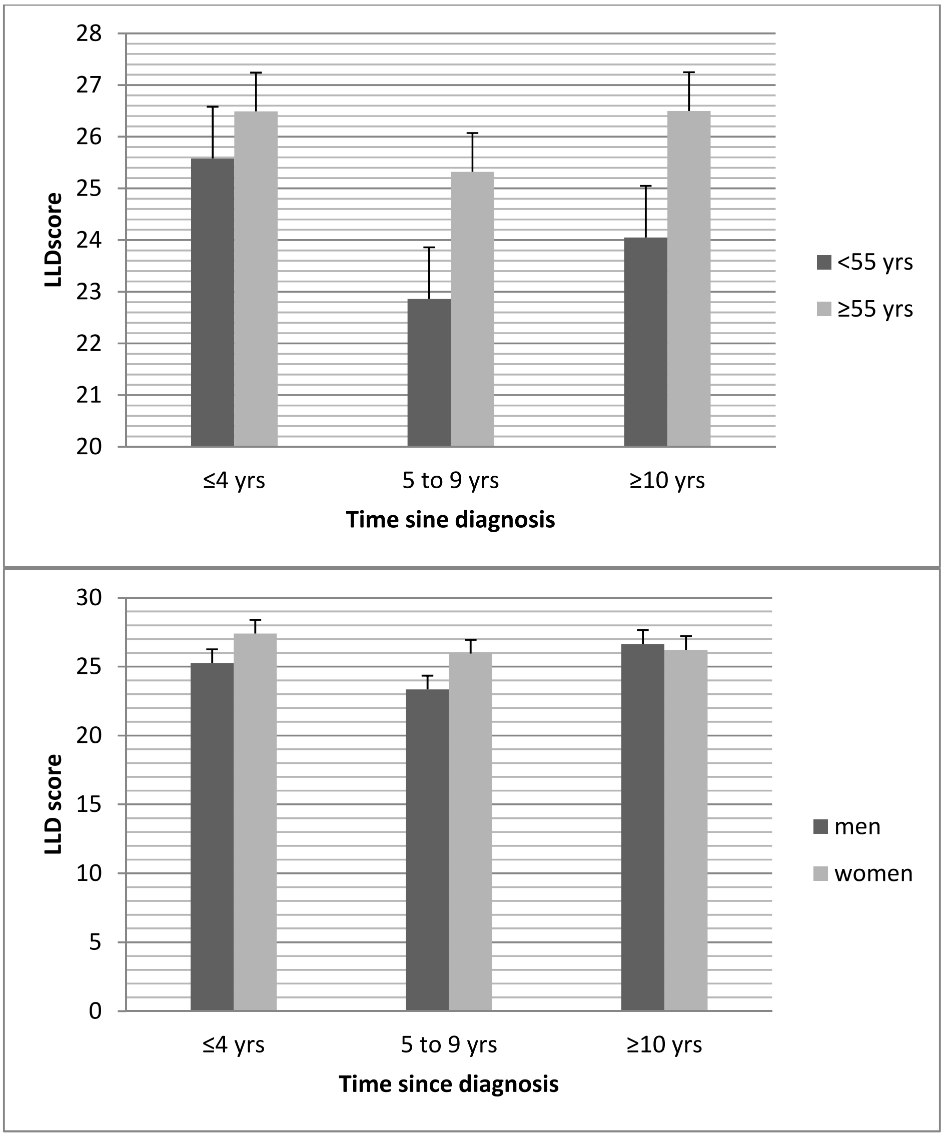

Figure S1: Mean LLD score according to age and sex category among GI cancers survivors stratified by time since diagnosis, Lifeline Study (2006-2009).

Abbreviations: GI, gastrointestinal**;** LLD, life Line diet.

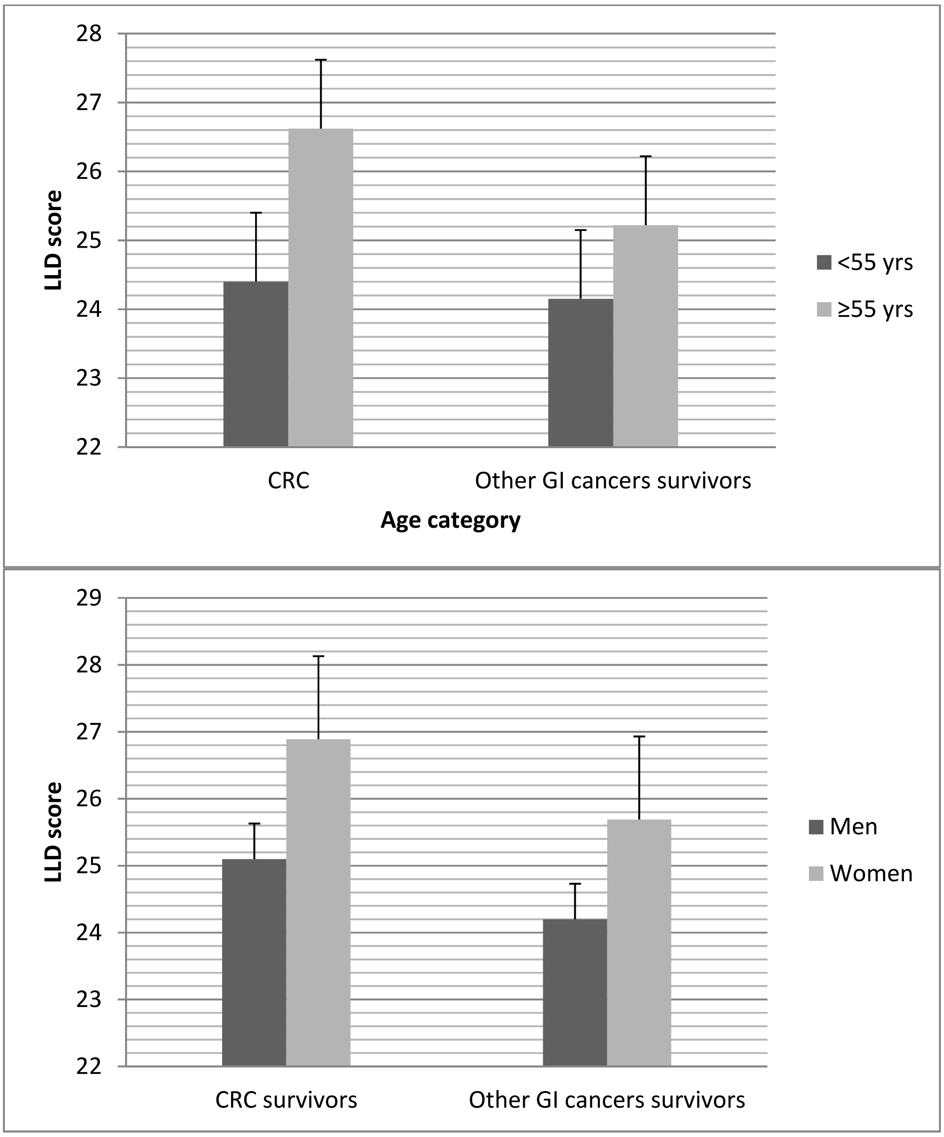

Figure S2. Mean LLD score according to age and sex category among GI cancers survivors stratified by tumour site, Lifeline Study (2006-2009).
